# Correlation Analysis of Peripheral Blood Inflammatory Cell Markers With Obesity in Chronic Schizophrenia Patients

**DOI:** 10.31083/AP52507

**Published:** 2026-09-07

**Authors:** Lili Wu, Xing Wei, Mengyun Zhang, Chengbing Huang, Wenjie Shi

**Affiliations:** ^1^Department of Psychiatry, Huai’an No.3 People’s Hospital, 223001 Huai’an, Jiangsu, China; ^2^Department of Psychiatry, Huai’an Xin’an Hospital, 223200 Huai’an, Jiangsu, China

**Keywords:** neutrophil-to-lymphocyte ratio (NLR), obesity, platelet-to-lymphocyte ratio (PLR), schizophrenia, systemic immune-inflammation index (SII)

## Abstract

**Background:**

This study investigated the association between obesity and peripheral blood inflammatory cell markers, namely the systemic immune-inflammation index (SII), neutrophil-to-lymphocyte ratio (NLR), and platelet-to-lymphocyte ratio (PLR), in patients with chronic schizophrenia.

**Methods:**

A total of 220 hospitalized patients with schizophrenia were enrolled. Obesity was assessed using body mass index (BMI), calculated according to standardized procedures. Participants were categorized into an obesity group (n = 86; BMI ≥28.0 kg/m^2^) and a non-obesity group (n = 134; BMI 18.5–24.0 kg/m^2^). Levels of SII, NLR, PLR, neutrophil count, lymphocyte count, and platelet count were measured and compared between the two groups. Correlations between SII, NLR, PLR, and obesity were subsequently analyzed.

**Results:**

Compared with the non-obesity group, the obesity group exhibited significantly higher SII values, lymphocyte counts, platelet counts, and suicide risk scores. BMI was positively correlated with SII. In addition, NLR was positively correlated with suicide risk scores.

**Conclusions:**

Patients with chronic schizophrenia and obesity exhibit elevated SII, lymphocyte counts, platelet counts, and suicide risk. SII may serve as a novel peripheral inflammatory marker for predicting obesity in this patient population.

## Main Points

1. In chronic schizophrenia, obesity was associated with elevated peripheral inflammatory markers and higher suicide risk; obese patients had significantly higher systemic immune-inflammation index (SII), lymphocyte counts, platelet counts, and suicide risk scores than non-obese patients, suggesting SII may be a potential inflammatory marker for obesity risk.

2. Body mass index (BMI) was positively correlated with SII; within the obesity group, neutrophil-to-lymphocyte ratio (NLR) was positively correlated with suicide risk scores, suggesting NLR may aid suicide risk assessment in obese patients with chronic schizophrenia.

3. These findings support an association among chronic low-grade inflammation, obesity, and suicide risk in chronic schizophrenia, although causality cannot be inferred.

## 1. Introduction

Schizophrenia (SZ) is a severe mental disorder that typically manifests during adolescence or early adulthood, characterized by core deficits across five symptom clusters affecting cognition, thought, and emotion. Obesity is a highly prevalent comorbidity in SZ, with rates approximately four times higher than in the general population. This elevated risk is attributed to multiple factors, including long-term antipsychotic use (particularly second-generation agents), sedentary behavior, poor dietary habits, physical inactivity, and adverse childhood experiences [1,2].

You and Shu [3] reported a metabolic syndrome prevalence of 45.4% among long-term hospitalized patients with chronic SZ, with rates of 48.4% in males and 41.9% in females. Obesity is associated with impaired immune function, a state potentially linked to systemic chronic immune inflammation involving the pancreatic islets, hypothalamus, liver, and gastrointestinal tract [4]. Unlike classic inflammation characterized by “redness, swelling, heat, and pain”, obesity is associated with a low-grade, chronic activation of the innate immune system, disrupting metabolic homeostasis—a state termed “chronic low-grade inflammation” [5]. Traditional markers such as complement component 3 (C3), complement component 4 (C4), C-reactive protein (CRP), interleukin-6 (IL-6), and tumor necrosis factor-α (TNF-α) have been implicated in this process [6,7].

In 2014, Hu et al. [8] proposed the systemic immune-inflammation index (SII), defined as peripheral platelet count (PLT) × neutrophil count/lymphocyte count, which reflects an individual’s systemic immune-inflammatory status. Subsequent research has indicated that SII, neutrophil-to-lymphocyte ratio (NLR), and platelet-to-lymphocyte ratio (PLR) may be associated with the progression and prognosis of various chronic conditions, including cancer and cardiovascular disease [9,10]. Recently, these inflammatory indices have gained attention for their potential clinical relevance in SZ [11]. Studies have demonstrated altered NLR and PLR in patients with SZ [12]. However, the relationship between these indices and obesity in chronic SZ warrants further investigation. The present study evaluated inflammatory indices such as SII, NLR, and PLR to explore the association between inflammation and obesity in chronic SZ, thereby providing a clinical rationale for understanding obesity in SZ from an inflammatory perspective.

## 2. Materials and Methods

### 2.1 Study Participants

A total of 220 inpatients with chronic SZ were recruited from Huai’an No.3 People’s Hospital in Jiangsu Province between January and August 2023. Based on body mass index (BMI), 86 patients (BMI ≥28.0 kg/m^2^) were assigned to the obesity group, and 134 patients (BMI between 18.5 kg/m^2^ and 24.0 kg/m^2^) were assigned to the non-obesity group. Participants with BMI between 24.0 kg/m^2^ and 28.0 kg/m^2^ (overweight) were excluded to maximize the phenotypic contrast between obese and normal-weight individuals as a narrow-phenotyping approach, and to reduce potential confounding from the intermediate inflammatory profiles of the overweight category. Furthermore, because this study was conducted in a Chinese Han population, the obesity threshold was defined as BMI ≥28.0 kg/m^2^ according to the Chinese guidelines for adult obesity (WS/T 428-2013), in accordance with local clinical practice, rather than the World Health Organization (WHO) criterion of ≥30.0 kg/m^2^ [13].

Inclusion Criteria: (1) age ≥18 years; (2) diagnosis meeting ICD-10 criteria for SZ; (3) illness duration ≥5 years; (4) no use of immunosuppressants, antibiotics, or similar medications within the preceding three months.

Exclusion Criteria: (1) other severe mental disorders or intellectual disability; (2) comorbid immune system disorders, diabetes, malignancies, cerebral infarction, or other serious somatic illnesses; (3) pregnancy or lactation.

### 2.2 Collection of General Information

A standardized demographic questionnaire was used to collect data, including study ID, name, sex, occupation, age, education level, marital status, illness duration, weight, height, and BMI.

### 2.3 Blood Parameter Analysis

Venous blood (5 mL) was collected from each participant, after an overnight fast, between 06:00 and 07:00 h on the same day as anthropometric measurements. A Mindray BC-7500CS automated hematology analyzer was used to determine complete blood counts, including lymphocyte count (L), neutrophil count (N), and PLT. SII, PLR, and NLR were calculated as follows: SII = PLT × N / L; PLR = PLT / L; NLR = N / L.

### 2.4 Suicide Risk Assessment

Suicide risk was assessed using the Nurses’ Global Assessment of Suicide Risk (NGASR), a validated 15-item scale that evaluates risk factors including suicidal ideation, plans, past attempts, psychiatric diagnosis, hopelessness, and social support. Total scores range from 0 to 25, with higher scores indicating greater suicide risk.

### 2.5 Statistics

Data were analyzed using SPSS Statistics (version 19.0, IBM Corp., Armonk, NY, USA). Continuous variables were compared using independent samples *t*-tests, and categorical variables were analyzed using the χ^2^ test. Pearson correlation analysis was performed to examine relationships between BMI and variables including SII, NLR, and PLR. Correlation analyses were conducted both within the obesity group separately and across all 220 participants combined. A two-tailed *p* ≤ 0.05 was considered statistically significant.

## 3. Results

### 3.1 Demographic and Clinical Characteristics

The study included 220 participants (86 in the obesity group and 134 in the non-obesity group). No significant differences were observed between the groups regarding age, sex, education level, or illness duration (*p* > 0.05 for all) (Table 1).

**Table 1.** **Comparison of demographic and biological parameters between the obesity and non-obesity groups**.

Variable		Non-obesity group (n = 134)	Obesity group (n = 86)	t/χ^2^ value	*p*
Age (years)		49.10 ± 10.46	46.49 ± 10.53	1.800	0.073
Sex, n (%)				3.903	0.066
	Male	88 (65.7%)	45 (52.3%)		
	Female	46 (34.3%)	41 (47.7%)		
Education (years)		9.10 ± 3.05	9.72 ± 3.20	1.428	0.155
Illness duration (years)		21.74 ± 10.76	19.50 ± 9.31	1.580	0.115
SII		339.89 ± 181.89	479.76 ± 332.86	3.570	<0.001
PLR		135.60 ± 53.99	137.51 ± 59.72	–0.246	0.806
NLR		2.14 ± 1.07	1.98 ± 0.96	1.070	0.286
Lymphocyte count (×10^9^/L)		1.68 ± 0.55	1.89 ± 0.67	–2.480	0.014
Neutrophil count (×10^9^/L)		3.35 ± 1.71	3.45 ± 1.41	–0.462	0.645
Platelet count (×10^9^/L)		209.63 ± 62.75	236.15 ± 76.52	–2.800	0.005
Suicide risk score		2.87 ± 0.89	4.78 ± 1.65	–11.110	<0.001

Note: Data are presented as mean (SD) or n (%). Statistical comparisons: independent-samples *t*-test for continuous variables; χ^2^ test for categorical variables. Abbreviations: SII, systemic immune-inflammation index; PLR, platelet-to-lymphocyte ratio; NLR, neutrophil-to-lymphocyte ratio; n, number of participants.

### 3.2 Comparison of SII, NLR, and Other Parameters Between Groups

The obesity group had significantly higher SII, lymphocyte counts, platelet counts, and suicide risk scores than did the non-obesity group (*p* < 0.05). No significant differences were found between the groups for NLR, PLR, or neutrophil count (*p* > 0.05) (Table 1).

### 3.3 Correlation Analysis of BMI With SII, NLR, and PLR in the Obesity Group

When analyzing all 220 participants combined, BMI showed a significant positive correlation with SII (r = 0.267, *p *= 0.018). Within the obesity group alone, BMI also demonstrated a significant positive correlation with SII (r = 0.284, *p* = 0.015) (Table 2). Both analyses supported the association. No significant correlations were found between BMI and NLR or PLR in either analysis. Furthermore, NLR was positively correlated with suicide risk scores (r = 0.242, *p* = 0.025).

**Table 2.** **Correlation analysis of various parameters with BMI in the obesity group**.

Parameter	Correlation coefficient (r)	*p*
Age	–0.107	0.369
Illness duration	–0.029	0.807
Neutrophil count	0.219	0.063
Lymphocyte count	–0.054	0.647
Platelet count	0.218	0.064
SII	0.284	0.015
NLR	0.128	0.280
PLR	0.157	0.183
Suicide risk score	–0.187	0.114

Note: Pearson correlation analysis. SII indicates a weak positive correlation with BMI. BMI, body mass index.

## 4. Discussion

Our study focused on the inflammatory mechanisms underlying obesity in chronic SZ, a common comorbidity linked to long-term antipsychotic therapy, unhealthy lifestyles, and early adverse events [14]. Although histaminergic and serotonergic signaling are known to drive antipsychotic-related weight gain, immune-inflammatory pathways have emerged as critical contributors to metabolic dysregulation in this population [15]. Obesity in psychiatric patients is not accompanied by overt acute inflammatory symptoms, but rather manifests as a persistent, low-grade activation of innate immunity that impairs metabolic balance—an established pathophysiological state known as chronic low-grade inflammation [16]. Conventional inflammatory biomarkers such as C3, C4, CRP, IL-6, and TNF-α have long been used to evaluate this inflammatory status [17]. In comparison, newer integrated indices, including SII and NLR, offer a more convenient and cost-effective approach to assessing systemic immune-inflammation levels, and their clinical value in SZ has attracted increasing research attention [18].

Our findings indicated that patients in the SZ obesity group exhibited higher SII and lymphocyte counts than did those in the non-obesity group, suggesting elevated peripheral chronic low-grade inflammation. The positive correlation between BMI and SII—though weak in magnitude—further indicated that elevated SII may be a modest risk factor for obesity in this population. Thus, SII holds potential as a predictive marker for obesity risk in chronic SZ. Our results were consistent with those of Chen et al. [18], who reported altered inflammatory cytokine production by mononuclear cells from obese SZ patients, and with those of Boozalis et al. [19], who found significant correlations between BMI and high-sensitivity CRP in SZ. However, Simpson et al. [20] noted that inflammatory and oxidative responses may occur independently of obesity in antipsychotic-treated SZ patients, suggesting a complex interplay.

Research has indicated that chronic low-grade inflammation involves senescent T cells, which adopt a senescence-associated secretory phenotype and release pro-inflammatory cytokines [21]. Chronic inflammation in obesity was proposed to lead to immune dysfunction or immunosenescence, resulting in the accumulation of these senescent T cells [20]. Similarly, Shirakawa and Sano [22] suggested that obesity may induce immunosenescence, impairing innate immune responses such as neutrophil and macrophage activation, and natural killer (NK) cell cytotoxicity. Conversely, other studies posited that obesity-related inflammation might trigger autoreactive or hyperactive T-cell responses, potentially modulated by PD-1 [23]. Yoshida et al. [24] demonstrated that a CD153 vaccine administration reduced senescent T cells in the visceral adipose tissue of obese mice and improved insulin resistance. Therefore, targeting senescent T cells presents a promising therapeutic strategy for obesity-related metabolic disturbances from an anti-inflammatory perspective.

Our study also found higher suicide risk scores in the obesity group, indicating that obesity may be associated with an increased suicide risk in chronic SZ. The positive correlation between NLR and suicide risk scores suggested that higher NLR levels might be linked to higher suicide risk in obese patients with chronic SZ. This was consistent with a previous study reporting that elevated NLR was significantly associated with increased suicide risk and could serve as a promising peripheral biomarker for suicidal behavior [25]. Similarly, Kachouchi et al. [26] reported associations between CRP and quality of life in SZ, and Şahpolat et al. [27] found elevated NLR and PLR in first-episode psychosis patients, suggesting that inflammatory disturbances are present early in the disease course. Collectively, NLR may serve as a valuable and accessible biomarker for assessing suicide risk in obese patients with chronic SZ, aiding in clinical risk stratification.

### Limitations

The present study had several limitations. First, we did not include a non-SZ control group, which limited our ability to determine whether the observed inflammatory alterations were specific to obesity within SZ or reflect SZ-related inflammatory processes in general. Second, we excluded individuals with a BMI between 24 and 28 (overweight), which reduced generalizability to this intermediate group. Third, the obesity definition (BMI ≥28 kg/m^2^) followed Chinese guidelines and may not have been directly comparable to studies using WHO criteria (BMI ≥30 kg/m^2^). Fourth, we did not collect data on antipsychotic medication types, dosages, or treatment duration, which may independently influence both inflammatory markers and body weight. Fifth, we did not include a symptom severity score (e.g., Positive and Negative Syndrome Scale); this could have confounded associations between inflammation and suicidality. Future research should involve multi-center studies with larger, matched samples, including healthy controls and comprehensive medication records, to validate and extend these findings.

## 5. Conclusions

Patients with chronic SZ and obesity exhibited elevated peripheral inflammatory markers (SII, lymphocyte count, platelet count) and higher suicide risk. The observed correlations between BMI and SII, and between NLR and suicide risk, suggested that these indices could be useful for comprehensive clinical assessment. SII may serve as a modest but novel predictor of obesity risk in this population.

## Data Availability

The data that support the findings of this study are not publicly available due to privacy and ethical restrictions, but are available from the corresponding authors upon reasonable request.
